# Challenges and opportunities in the diagnosis and treatment of early-onset psychosis: a case series from the youth affective disorders clinic in Stockholm, Sweden

**DOI:** 10.1038/s41537-023-00427-z

**Published:** 2024-01-03

**Authors:** Mathias Lundberg, Peter Andersson, Johan Lundberg, Adrian E. Desai Boström

**Affiliations:** 1 Stockholm Health Care Services, Region Stockholm, Stockholm, Sweden; 2 The Affective Disorders Clinic, Child and Adolescent Psychiatry, Stockholm Health Care Services, Region Stockholm, Stockholm, Sweden; 3 Department of Clinical Science and Education, Södersjukhuset, Internal Medicine, Karolinska Institutet, Stockholm, Sweden; 4 Department of Clinical Neuroscience/Psychology, Karolinska Institutet, Stockholm, Sweden; 5 Centre for Psychiatry Research, Department of Clinical Neuroscience, Karolinska Institutet, & Stockholm Health Care Services, Region Stockholm, Karolinska University Hospital, SE-171 76 Stockholm, Sweden; 6 Department of Clinical Sciences/Psychiatry, Umeå University, Umeå, Sweden

**Keywords:** Psychosis, Psychiatric disorders

## Abstract

Early-onset psychosis is linked to adverse long-term outcomes, recurrent disease course, and prolonged periods of untreated illness; thus highlighting the urgency of improving early identification and intervention. This paper discusses three cases where initial emphasis on psychosocial treatments led to diagnostic and therapeutic delays: (1) a 15-year-old misdiagnosed with emotionally unstable personality disorder and autism, who improved on bipolar medication and antipsychotics; (2) another 15-year-old misdiagnosed with autism, who stabilized on lithium and antipsychotics, subsequently allowing for gender dysphoria evaluation; (3) a 9-year-old autistic boy incorrectly treated for ADHD, who recovered with appropriate antipsychotic treatment. These cases illuminate the vital importance of adhering to a diagnostic hierarchy, prioritizing diagnostic utility, and conducting longitudinal evaluations to facilitate early targeted treatment of psychotic symptoms in early-onset psychosis. Adherence to such strategies can minimize delays in managing early-onset psychosis and improve long-term prognoses.

## Introduction

Early-onset psychosis (EOP), a broad clinical concept referring to the onset of psychosis in conjunction with schizophrenia spectrum, affective and other non-affective psychotic disorders before the age of 18^1^ impacts an estimated 11–18% of individuals diagnosed with schizophrenia spectrum disorders^2^. The age of onset is a significant predictor of long-term outcomes, with earlier onset associated with more frequent hospitalizations, relapses, and poorer occupational and social functioning^3^. Despite its significance, current EOP management strategies remain suboptimal, leading to unfavorable long-term outcomes in 50–60% of cases^4^. There is a clinical imperative to minimize the duration of untreated psychosis^5^, especially as EOP is associated with considerable delays in initiating antipsychotic treatment^6^. Furthermore, each recurrent episode of schizophrenia increases the risk of treatment resistance^7^.

The UN Committee on the Rights of the Child (CRC) recently advised Sweden to prioritize psychosocial interventions for child mental health, citing concerns about pharmaceutical overuse^8^. In a separate interview with Sweden’s newspaper of record, Dagens Nyheter, the chair of the CRC further clarified that medications should only be used for child and adolescent mental health when all other alternatives have been exhausted^9^. While well-intentioned, this policy recommendation may restrict treatment options for youth with EOP. Importantly, this advice appears to diverge from evidence indicating that timely administration of advanced medico-psychiatric treatments (e.g., Clozapine) can contribute to reducing suicide deaths in male adolescents^10^.

Diagnosis of EOP is already challenging due to the frequent occurrence of psychotic-like symptoms in other psychiatric disorders within this age group^11^. Further complexity arises from research showing that differing theoretical frameworks for understanding severe mental illness in youth can result in varied treatment approaches for the same symptomatology, demonstrated to profoundly impact patients’ chances of recovery^12^. Psychotic disorders/schizophrenia are further known to have overlapping symptoms, and increased frequency in comorbidity can be a diagnostic and therapeutic challenge in clinical practice. This may be of particular relevance to youth and EOP. There are marked regional variations in autism spectrum disorder (ASD) diagnoses among females aged 10-19—particularly with prevalence rates in Stockholm outpacing those in Gothenburg and Skåne by approximately threefold—raising questions about the overshadowing of undiagnosed severe mental disorders^13^. The clinical trajectory of youth initially diagnosed with ASD in Stockholm may serve as a critical case study with global implications. It highlights the risks of overshadowing other severe mental disorders when there’s an undue focus on ASD in youth psychiatric care^14^, especially in light of U.S. case reports that emphasize treatable yet often overlooked episodes of severe mental illness in autistic youth^15^.

This series features case studies from the Child and Adolescent Affective Disorders Clinic in Region Stockholm, established in September 2022. The clinic, staffed by two associate professors with extensive expertize in severe mental illness in youth, presents three cases where severe mental illnesses were initially overlooked in favor of psychosocial treatments. In light of the clinic’s patient profile—youth with severe symptoms and high suicide risk—the diagnostic approach prioritizes utility over validity^16^. For example, patients who display severe impairment and are deemed high-risk for underlying bipolar disorder may be diagnosed and treated accordingly, even when their symptoms have been previously interpreted otherwise. This approach allows for urgent and effective treatment interventions and is clearly communicated to, and consented by, patients and their legal guardians.

## Patient 1. navigating complexities in treatment-resistant depression with psychotic features: a bipolar disorder perspective in an adolescent

### Case presentation

A 15-year-old Caucasian female patient comes from a well-functioning, upper-middle-class family with a history of schizophrenia in her grandmother. She also has a medical history of insulin-treated Type 1 Diabetes Mellitus (DM). She experienced her first depressive episode at the age of 13– which was conservatively treated with psychoeducation and supportive counseling. Prior to this, her childhood and schooling progressed without any psychopathological indications. Clinical interviews, anamnesis, and lab results showed no evidence of substance use or alcohol abuse. Furthermore, all drug screenings conducted upon admission to inpatient services have consistently been negative throughout her clinical course. During her adolescence, urine drug tests and blood tests of transaminases sensitive to alcohol consumption have repeatedly confirmed the absence of substance or alcohol abuse. However, her condition has deteriorated over the past two years: she has become socially isolated, engaged frequently in self-harm, consistently missed school, and made several severe suicide attempts that required intensive care.

### Treatment onset and functional deterioration

The severity of the patient’s condition led her parents to set aside their professional commitments to focus solely on her care and well-being. Her treatment plan consisted of regular appointments with youth psychiatrists and sessions centered around Cognitive Behavioral Therapy (CBT). Following a suicide attempt shortly after initiating conservative treatment for MDD, she received a Children’s Global Assessment Scale (CGAS) score of 50. Initial pharmacological interventions included aripiprazole (with a maximum dose of 10 mg/day), and melatonin was administered for sleep issues before being replaced by propiomazine. Promethazine and sertraline (25 mg/day) were prescribed to address her affective symptoms. It’s worth noting that sertraline coincided with clinical observations of hyperactivity and intensified suicidal thoughts, leading to its discontinuation.

### Reevaluation: unresponsive to SSRIs and indicators of autism

Subsequently, the patient’s lack of response to low-dose SSRIs and ongoing self-harm necessitated a clinical reevaluation. This reassessment was initiated by a clinician who first encountered her in the psychiatric emergency department following a suicide attempt. Despite her previously unremarkable functional history and current depressive symptoms, several potential signs of autism were observed, including rigidity, lack of facial expression, reticent speech, and low social functioning. The clinician speculated that camouflaging behaviors in her early life might have masked these autistic traits. Additionally, her suicidality was attributed to emotional dysregulation, which was thought to be secondary to a lack of adequate social adaptations at home and school for her suspected autism, thereby causing stress and dysregulation. Her self-harm was interpreted as an autistic ‘locking’ onto the concept, likely intensified by the inherent rigidity associated with autism. A subsequent neuropsychiatric assessment revealed a standard-range IQ but with specific deficits in visuospatial abilities. Autism Diagnostic Observation Schedule (ADOS)^17^ testing led to a diagnosis of mild autism spectrum disorder, and she was also diagnosed with Reaction to Severe Stress, both according to ICD-10 criteria.

### Ongoing suicidality and self-harm amidst autism interventions

However, despite undergoing autism-focused interventions—comprising psychoeducational therapy for both the patient and her parents, delivered by specialized neuropsychiatric habilitation services, as well as psychotherapy tailored to her autistic traits—her suicidality persisted. Frequent suicide attempts and escalating severity of self-harm episodes led to multiple short-term hospitalizations. During these stays, the emphasis was on stress reduction within a low-stimulus environment, accompanied by psychosocial preventive measures against self-destructive behavior. Although these interventions led to a decrease in both the frequency and intensity of self-harm and suicidal thoughts, frequent relapses were observed shortly after each discharge.

### Diagnostic reassessment and treatment adaptions

During this period, Fluoxetine was introduced to manage anxiety. However, a rapid dose escalation was temporally correlated with worsening mood swings and a resurgence of persistent self-harm. As a result, her care was transitioned to a team specializing in Dialectical Behavior Therapy (DBT). She met five of the nine diagnostic criteria for Emotionally Unstable Personality Disorder (EUPD). The difficulties in ascertaining this condition in conjunction with the autistic traits were noted as a diagnostic challenge. Nevertheless, the EUPD diagnosis was selected to facilitate targeted DBT treatment for her self-harming behavior.

Despite attending 59 DBT sessions over an eight-month period—a regimen both she and the treating clinician described as beneficial—the frequency and severity of her suicidal behaviors escalated. This prompted a consultation with a clinic specializing in bipolar and psychotic disorders. The clinic referred the patient back for continued DBT treatment, citing an absence of manic episodes and suggesting a comorbid autism/EUPD diagnosis as more plausible. DBT sessions resumed for a brief period.

A particularly alarming incident involved a suicide attempt by insulin overdose. This led the treating clinician on the DBT team to diagnose the patient with Bipolar Disorder Type II. Treatment with Lamotrigine was initiated at 25 mg per day, with dose increases of 25 mg every two weeks, reaching a maintenance dose of 75 mg. Subsequently, the patient was referred to the Affective Disorders Clinic.

### Diagnostic shift: considering bipolar II with psychotic features

The initial assessment of the patient revealed significant challenges, such as social withdrawal, self-injurious behavior, suicidal ideation, and familial discord. The patient’s limited social engagement and considerable functional decline were substantiated by a Children’s Global Assessment Scale (CGAS) score of 35. A reevaluation of an autism diagnosis was prompted by the absence of documented functional impairments before the age of 10. The psychiatric history review uncovered a pattern of emotional instability, particularly a rebound from a depressive state necessitating hospitalization. Following discharge, the patient displayed mood improvement, evidenced by meticulous diabetes management, enhanced social engagement, and the absence of depressive or anxiety symptoms. Yet, this period of amelioration was short-lived, as the patient soon relapsed into severe depression, necessitating further psychiatric care.

The patient’s non-response to Dialectical Behavior Therapy (DBT), symptom aggravation post-selective Serotonin Reuptake Inhibitor (SSRI) treatment, positive screenings on both the Mood Disorders Questionnaire (MDQ)^18^ and the Child Mania Rating Scale-Parent Version (CMRS-P)^19^, along with mood-congruent auditory hallucinations promoting self-harm, all pointed towards Bipolar II Disorder with psychotic features as a differential diagnosis. This diagnosis was subsequently confirmed by the leading clinician using the Longitudinal, Expert, All Data (LEAD) standard (refer to Supplemental Table 1 for detailed diagnostic criteria). Noteworthy is the absence of any indicators of a thought disorder or disorganized behavior throughout the evaluations.

### Symptom improvement post-lithium and psychotic feature revelation

Treatment with Lithium was promptly initiated, and weekly blood tests facilitated rapid titration to a predetermined blood concentration of 0.8 mmol/L, reflecting the severity of the patient’s psychiatric symptoms. In the first month following Lithium initiation, the patient showed marked improvement, becoming more communicative, presenting with reduced self-harm, and significantly diminishing the intensity of suicidal thoughts. As her depressive symptoms receded and communicability improved, the patient disclosed pre-existing psychotic symptoms, including tactile hallucinations of insects crawling under her skin and paranoid ideation. She indicated that these symptoms had been present at similar levels of intensity and frequency prior to starting Lithium therapy.

### Pharmacotherapy optimization antipsychotic management

To further address these symptoms, Risperidone was initiated at 0.5 mg/day, with rapid increments of 0.5 mg every third day, targeting a dosage of 4 mg/day. Low-dose Olanzapine (5 mg) was also added, influenced by case studies suggesting that it can mitigate the common side effects of Risperidone and thereby enhance treatment tolerability^20^. During the titration phase for Risperidone, the patient experienced sedation and excessive daytime sleepiness, both of which fully resolved upon reaching the target dose. No other medication-related side effects were reported.

### Clinical improvement and return to daily activities

The initiation of adjunctive antipsychotic treatment coincided with a dramatic clinical improvement. The patient became more proactive in her treatment, actively seeking help and communicating openly about her feelings and urges. Notably, her self-harming behavior ceased entirely, and she no longer displayed suicidal ideation. This significant treatment progress enabled her transition from daily psychiatric visits to spending time abroad on vacation with her family, and she has also recently returned to school.

## Patient 2. unveiling the interplay of gender dysphoria, psychosis, and bipolar disorder: challenges and insights in adolescent care

### Case presentation

A 15-year-old Caucasian, who was assigned female at birth and is reported to have been identifying as male for the past 3 years, has successfully undergone an official gender registration change. The patient demonstrated giftedness and artistic talent during childhood, and had no previous engagements with Child and Adolescent Mental Health Services. Onset of anxiety and depression emerged around the ages of 9-10 and progressively intensified. A reported traumatic episode involving peer-related sexual harassment was initially attributed to catalyzing these mental health issues. Prior to symptom onset, the patient maintained supportive relationships, was socially active in an age-appropriate manner, and showed no indications of substance abuse or inheritable severe mental disorders.

### Initial treatment and neuropsychiatric assessment

Following the initial evaluation, trauma-focused CBT was implemented in tandem with adjunctive hydroxizine, melatonin, and sertraline (Sertrone 25–50 mg) to manage sleep disturbances and depressive manifestations. Worsening symptoms, marked by escalated anxiety, self-harm, and reported auditory hallucinations urging self-destruction, necessitated a referral to the psychosis and bipolar disorder clinic (i.e. the same clinic Patient 1 was referred to). However, the referral was declined in favor of a neuropsychiatric evaluation and low-dose aripiprazole (5 mg) administration. This intervention aimed to address auditory hallucinations, then believed to be stress-induced symptoms linked to potential undiagnosed autism. The subsequent neuropsychiatric evaluation resulted in a diagnosis of atypical autism, alongside suspected Emotionally Unstable Personality Disorder (EUPD) traits. Cognitive testing showed above-average abilities.

### Escalating symptoms and treatment challenges

Six months post-initial psychiatry interaction, the patient made a suicide attempt by slashing their wrists in a bathtub after leaving a farewell note. This was followed by a second attempt via paracetamol overdose a month later. Outpatient assessment revealed an APSS score of 2.5, suggesting potential risk for a psychotic disorder. However, an alternative explanation was proposed that these symptoms could stem from dissociative causes, which guided initial interventions. Despite ongoing treatment, the patient remained largely unresponsive for the initial 18 months, with persistent CGAS scores below 50. Notably, to avoid potentially reinforcing EUPD-linked suicidal behaviors, inpatient services were frequently denied by emergency services after suicide attempts and severe self-harm events, and parents were instructed to not overly fixate on the patient’s self-destructive tendencies.

### Reassessment at specialized clinic

A subsequent reevaluation at the clinic specializing in psychosis and bipolar disorders scrutinized the presence of auditory hallucinations and potential hypomanic indicators. It was inferred that the presented symptoms were more congruent with atypical autism and probable EUPD. Features considered dissociative included the patient’s perception that compulsive, self-harming thoughts were implanted in their mind, which apparently bolstered EUPD suspicions. A personality assessment focusing on EUPD and Dialectical Behavior Therapy (DBT) was recommended.

### Navigating diagnostic and treatment complexities

During ongoing assessments conducted by the DBT team—prior to the initiation of any DBT treatment—the team identified a need for a more comprehensive assessment to either confirm the existence of EUPD traits or to determine if the symptoms could be more accurately described as a combination of autism, bipolar disorder, and post-traumatic stress disorder. Given the severity of the patient’s symptoms, treatment was initiated with low-dose sertraline (25–50 mg/day) to manage her depressive symptoms. Adjunctive lamotrigine treatment was also started at a dose of 25 mg/day, increasing by 25 mg every two weeks to a final dosage of 75 mg/day, with the aim of managing any potentially hypomanic manifestations. Despite these measures, the patient’s frequent self-injurious and parasuicidal behavior continued and remissions of psychiatric symptoms were inadequate. Concurrently, the patient was referred to the affective disorders clinic for an in-depth assessment.

Despite urgently needing it, the patient had been consistently denied a referral for a gender dysphoria assessment at a specialized clinic. The refusal was based on the argument that the severity of the patient’s symptoms made such an assessment inappropriate at the time.

### Bipolar disorder consideration and treatment adjustment

During the initial evaluation at the Affective Disorders Clinic, the patient was administered the Mood Disorders Questionnaire (MDQ)^18^, the Adolescent Psychotic-Like Symptom Screener (APSS)^21^, and the Child Mania Rating Scale-Parent Version (CMRS-P)^19^, with scores of 13, 3, and 24, respectively. These scores indicated a positive screening for bipolar disorder with psychotic features. The findings were corroborated by the Mini International Neuropsychiatric Interview for Children and Adolescents (MINI-KID). The patient also exhibited symptoms of atypical depression, including hypersomnia, a sensation of leaden paralysis, psychomotor retardation, and excessive guilt.

A review of the patient’s history revealed episodes of irritable mania, marked by heightened energy, increased activity, and risk-taking behaviors, with a notably more talkative demeanor and pressured speech. A comprehensive evaluation, integrating psychiatric assessment and detailed anamnesis from the patient and parents, led to the diagnosis of Bipolar Disorder Not Otherwise Specified (NOS) based on the LEAD procedure. Consequently, sertraline was discontinued. Refer to Supplemental Table 2 for a complete diagnostic assessment.

### Rapid pharmacological impact and functional gains

Subsequent to this event, the patient engaged in self-harm with razors and sought emergency medical care. This prompted the need for enhanced clinical monitoring. A rapid titration regimen of Quetiapine XR was initiated: 100 mg on the first day, 200 mg on the second day, and 300 mg on the third day, supplemented with lithium. The intent was to transition solely to lithium therapy once a blood concentration of 0.8 mmol/l was reached. This dual pharmacological approach aimed to swiftly alleviate depressive symptoms while mitigating the risks of inducing hypomanic episodes and minimizing the potential for unwanted weight gain associated with long-term usage of Quetiapine XR. In the months that followed, there was a marked improvement in the patient’s affective stability, evidenced by the complete absence of any suicidal or self-harming behaviors requiring medical intervention. After transitioning to lithium monotherapy—titrated and confirmed to reach a concentration of 0.8 mmol/l within eight weeks—the patient’s CGAS scores consistently increased from a range of 42–48 to 55–65.

### Psychosocial gains and high school transition

Auditory hallucinations persisted even after mood symptoms had stabilized—centered on egosyntonic voices inciting self-harm and suicide - prompting the introduction of risperidone at a starting dose of 0.5 mg. The dose was increased to 1.5 mg over the course of 4.5 months, beginning 5.5 months after the initiation of lithium therapy. This treatment regimen led to the cessation of hallucinations over approximately five months and was accompanied by a marked functional improvement. This improvement was reflected in the Children’s Global Assessment Scale (CGAS) scores, which consistently rose from 60 at the clinical visit where risperidone was prescribed to 75 after five months of treatment. By the end of this period, the patient had shown significant functional gains, was planning to start high school, had integrated well within their family, and was socially active. They even secured summer employment. Additionally, a previously deferred assessment for gender dysphoria was reinitiated and was reported to have significantly reduced distress for both the patient and their family.

## Patient 3. reclaiming stability from disorganization: a medical journey to remission in a child with disorganized psychosis

### Case presentation

The patient, a 9-year-old Caucasian boy, was brought to the clinic when his father reached out. There was a documented history with psychiatric services due to a diagnosis at age 4 of atypical autism, intellectual disability, and language disorder with delayed language development. This was based on observed challenges in verbal and social abilities. The patient hails from an upper-middle-class background, with both parents having postgraduate qualifications. Notably, there’s no history of substance abuse or traumatic events. Prior of to the current episode, with adequate support from the family, the patient had been able to achieve relatively good functioning, with no aggression, self-injurious behavior or affective lability. The family does have a history of dyslexia, and a third-degree relative on the father’s side has autism. Still, there is no known hereditary risk for severe psychiatric illness.

Patient 3 had been attending a specialized class designed for children with autism. Although he had to repeat a year of preschool due to learning difficulties, he adapted reasonably well until a year before this clinical encounter. At that point, his functional abilities notably deteriorated, marked by aggressive, restless, and self-harming behaviors. His school performance also declined, and contact with the Child and Adolescent Mental Health Services increased. An updated neuropsychiatric evaluation was conducted, resulting in a diagnosis of severe intellectual disability, high-impairment autism, ADHD, and a behavioral disorder. Consequently, treatment with central stimulants commenced about nine months prior to the patient’s presentation at the Affective Disorders Clinic.

### Treatment initiatives and functional decline

The deterioration of the condition continued after initiation of central stimulant treatment, the patient’s condition deteriorated further with escalated outward aggression and reduced language skills. A subsequent introduction of a low dose of aripiprazole (5 mg/day) was followed by heightened motor activity, restlessness, and pronounced behavioral disturbances, including emotional agitation. The patient’s aggression, particularly towards his mother, escalated. Consequently, the father often had to relocate the patient to the family’s secondary apartment for play and respite. The situation worsened in the subsequent months, with increased violent behavior, self-harm, and significant school avoidance.

### Suspicion of psychosis with disorganized symptoms

ML’s involvement as a senior consultant began about a year after the patient’s decline, initiated when the patient’s grandfather acutely visited the clinic and demanded an assessment. Previous attempts to assess the patient in emergency services had been rendered impossible due to the patient’s highly agitated behavior. The grandfather’s descriptions, which characterized the patient’s behavior as aimless, fearful, impulsive, and marked by a significant reduction in previous functional abilities, led to preliminary suspicions of disorganized psychosis. Given the urgent need to prevent harm and disability, it was decided to initiate treatment without a direct face-to-face assessment. Based on the information available in the patient’s medical records, low-dose risperidone started at 0.5 mg and later increased to 1 mg, with the condition of close follow-up. Central stimulants were discontinued promptly. The first in-person consultation with the patient took place a few weeks after the new treatment had been initiated. While improvements in self-harming tendencies and outward aggression were reported, the patient remained notably disorganized during the clinical visit and showed clear signs of paranoid thinking; for instance, the patient went to the window, observed cars, and pondered aloud whether they were police cars, even asking the clinician if he was a police officer. Self-rating was not possible due to the patient’s clinical condition.

### Post-treatment behavioral and cognitive improvements

Four months post the antipsychotic treatment initiation, substantial improvements were observed. The patient’s language skills improved significantly, and disorganized behavior reduced considerably. By summer break, he resumed home-based school activities, with staff noting marked improvements compared to previous encounters. While the patient no longer exhibited overtly psychotic or intrusively disorganized behavior, some concentration challenges and subtle symptoms of thought disorders persisted.

### Progress toward baseline functionality

At present, the patient is on a 2 mg dosage of risperidone, with gradual increments planned to achieve the optimal therapeutic dose. The parents report that the patient’s functional ability, relative to his age, seems to be reverting to its baseline state (refer to Supplemental Text 1 for a detailed rationale on the decision to continue treatment on the suspicion of disorganized psychosis).

## Discussion

### Patient 1: the imperative of continuous diagnostic reassessment in adolescents

Firstly, while ‘camouflaging’ in autism is acknowledged, attributing rapid clinical decline solely to neuropsychiatric issues can miss urgent conditions. Prioritizing diagnostic validity over clinical utility—by strict adherence to NICE guidelines that require evidence of manic episodes—may withhold crucial treatments like lithium. A hierarchical diagnostic approach that prioritizes manifestations of severe mental illness appears more suitable for adolescents with severe psychiatric presentations^22^. This is further exemplified by the initial misinterpretations of imperative auditory hallucinations as symptomatic of conditions like autism or emotionally unstable personality disorder (EUPD). In contrast, a more hierarchical diagnostic approach would prioritize considering these hallucinations as potential indicators of psychosis, unless robust evidence suggests alternative explanations. This perspective underscores the critical need for nuanced diagnostic algorithms that recognize the high stakes of early intervention in achieving favorable long-term outcomes.

Secondly, it was the patient’s lack of response to a low dose of Sertraline (25 mg) for depression, rather than any adverse drug reaction, that prompted a reevaluation of the initial diagnosis from depression to autism. It’s crucial to note that in adult populations, Sertraline dosages below 50 mg/day have not shown a superior effect compared to placebo for treating MDD^23^. Although there is more limited data on adolescents, the non-response to this low dosage should not be the sole basis for a diagnostic shift, such as reclassifying the condition from depression to autism. Therefore, it’s important to exercise caution when interpreting the lack of response to low-dose SSRIs as diagnostic evidence.

Third, while self-harm may traditionally most be associated with EUPD and autism—this case demonstrates that untreated episodes of EOP may also introduce such behavior.

#### Therapeutic challenges

An eight-month period with 59 intense DBT sessions—reported by both patient and therapist as beneficial and crucial to prevent self-harm—coincided with increased suicidal behaviors; indicating that subjective reports of improvement may stem more from regular clinical interaction than effective symptom management. This has relevance to DBT-research practices, suggesting that subjective measures may not correlate with key outcomes, such as the frequency and intensity of suicidality in this case. It is worth noting that the ineffective application of DBT, could extend the duration of untreated illness. This is particularly concerning for severely mentally ill adolescents who require advanced psychiatric interventions.

#### Affective and psychotic symptom interplay

As mood symptoms receded, latent psychotic symptoms became evident. Addressing these was crucial for managing the patient’s suicidality, and clinicians may consider the possibility of latent psychotic symptoms in severe psychiatric presentations unresponsive to standard treatments.

This case accentuates the need for continual reassessment, especially when treatment resistance or new symptomology is observed. Given the stakes involved—sometimes life and death—meticulous attention to these factors is not just advisable but essential.

### Patient 2: differential diagnosis in youth with complex neuropsychiatric presentations

The second case shows that treating bipolar disorder with psychotic features can make gender dysphoria assessments possible. Prior to treatment, the patient had been denied such assessments due to the severity of their symptoms. Up to 25% of individuals with schizophrenia may also experience gender dysphoria^24,25^. The case confirms both conditions can co-exist, and gender dysphoria may persist post-psychotic treatment. Effective treatment of psychotic symptoms thus serves dual purposes: improving mental health and facilitating gender reassignment assessments.

Furthermore, this case underscores the importance of adhering to a hierarchical approach in the diagnostic process. Similar to case 1, the auditory hallucinations were initially considered to be symptoms of less severe disorders rather than signs of an ongoing psychotic process. It was not until structured screening tools suggested the possibility of psychosis, which was initially thought to indicate dissociation associated with Emotionally Unstable Personality Disorder (EUPD), that a reevaluation occurred. As with case 1, a significant remission of symptoms was only achieved after these experiences were reclassified as psychotic symptoms and treated with targeted antipsychotic medication.

### Patient 3: misdiagnosis and the primacy of severe mental disorders

This case emphasizes the need for thorough assessments in patients with rapidly worsening behavioral and cognitive symptoms. The symptom overlap between disorganized psychosis and comorbid ADHD/ASD further highlights the risks of not using a diagnostic hierarchy. Initial assessments focused on neuropsychiatric and behavioral comorbidities, potentially overlooking broader severe mental illnesses like psychosis. Such misdiagnoses can lead to ineffective treatments that exacerbate the condition; such as the prescription of central stimulants to a child with severe psychotic symptoms. In youth with acute episodes and significant functional impairments, clinicians may improve outcomes by focusing on possible severe mental illnesses rather than multiple, less severe conditions^22^. This approach requires deep understanding of neurobiology, cognitive processes, and symptom trajectory. Overall, the case stresses the importance of precise differentiation among these conditions, and suggests prioritizing treatment of severe mental disorders for better outcomes.

### Strengths and limitations

The efficacy of advanced psychiatric interventions in treating complex cases supports the validity of the selected treatment strategies. However, not all patients may respond similarly, and these treatments are not devoid of side effects. For instance, antipsychotics are linked with increased neurological and metabolic adverse effects in young patients^26,27^, though strategies exist to lessen these effects^20,27^. Hyperprolactinemia is a clinically significant adverse effect of risperidone treatment in both adults^28,29^ and, putatively, adolescents^30^. Clinical manifestations such as gynecomastia and galactorrhea represent potential concerns. In response, current evidence suggests that clinicians might consider adjunctive therapy with aripiprazole^31^ or, when clinically indicated, transition to an alternative antipsychotic^32^ as effective management strategies. In this clinic, continuous monitoring of this side effect is standard practice to ensure prompt and appropriate intervention. While lithium is proven safe and effective in the short term, the long-term effects and some observed adverse reactions necessitate ongoing clinical vigilance^33,34^. Registry studies suggest that lithium maintenance therapy correlates with reduced suicide rates and overall mortality in patients with high-risk bipolar disorder^35^, with additional evidence pointing to its neuroprotective properties^36^. It is crucial for clinicians to be skilled in conducting thorough risk-benefit analyses and in monitoring treatment responses vigilantly.

The precise prevalence of EOP in the general population has yet to be established. Nonetheless, its incidence is expected to be considerably greater in specialized clinical settings, especially among those severe cases that show resistance to conventional treatments. These instances are a recognized challenge within youth psychiatric services. Consequently, independent of the actual frequency of EOP, it is critical for youth psychiatric practices to possess the necessary expertize to identify and treat these patients effectively. This is akin to the responsibility of primary care professionals to adeptly detect and respond to less common presentations of chest pain, such as myocardial infarctions. For diagnostic clarification in complex clinical scenarios, hospitalization may be warranted to allow for rigorous observation by a multidisciplinary team. Such a team should follow a systematic and tiered approach to diagnosis to facilitate effective clinical management.

Clinical diagnosis is an evolving process, where initial hypotheses are honed based on the patient’s response to treatment. Such an iterative method is enlightening and often necessitates reevaluation of first impressions^37^. Integrating this dynamic approach into pediatric psychiatry practice has the potential to refine diagnostic pathways, accelerate the onset of effective treatments, and improve long-term patient outcomes.

## Conclusion

The presented cases provide a window into the complex landscape of adolescent mental health care, where the medical and psychosocial models converge and diverge. In all cases, suspicions of severe psychopathology were raised early-on; yet failed initially to materialize into targeted treatment. Early clinical assessments in these cases initially leaned towards explanatory models that attributed these experiences to conditions considered less severe, yet also less amenable to advanced psychiatric interventions. This misdirection underscores the need for a shift in diagnostic focus. Prioritizing a hierarchical perspective, along with longitudinal course and clinical utility—a concept previously emphasized as important in nosological conceptualization^38^ and diagnostic assessment^16^—over strict diagnostic validity, could be pivotal in the early recognition of severe illness states, such as EOP, in children and adolescents. For a summary of key clinical concepts illustrated by the cases and recommendations for diagnosis and treatment in pediatric psychiatry, please see Table 1 and Table 2. Affective and psychotic symptoms pertaining to each case are described in Table 3. For patient perspectives, please see Supplemental Text 2.

**Table 1:** Clinical decision-making and outcomes in pediatric and adolescent psychiatry cases.

Patient	Clinical situation	Premature clinical interpretation	Clinical action taken	Consequences
Patient 1	Non-responsive to low-dose SSRI	Interpreted as contraindicating depressive disorder	Initiation of neuropsychiatric assessment	Premature shift in diagnostic hierarchy favoring less severe conditions
	Patient and clinician perceiving progress in DBT treatment, but no decreased suicidal behavior	Continued DBT treatment assessed as indicated	Continuation of treatment	Extended duration of untreated mood disorder
Patient 2	Significant instability, pronounced self-injurious behavior, auditory hallucinations	Auditory hallucinations interpreted as part of neuropsychiatric condition with severe stress	Low dose (5 mg) aripiprazole treatment initiated; referral to neuropsychiatric assessment	Delayed diagnosis
	Structured assessment tools indicating possible psychosis (APSS score: 2.5), self-report of auditory hallucinations and invasive thoughts	Symptomatology interpreted as dissociative, linked to putative EUPD traits	Instructions to parents regarding non-responsiveness to self-injurious behaviors; continuation of EUPD assessments	Disregard for clinical needs of patient; delayed diagnosis
Patient 3	Rapid clinical deterioration with outward aggression, self-injury, restlessness; pre-existing neuropsychiatric symptomatology	Interpreted as indicating neuropsychiatric evaluation, resulting in ADHD diagnosis	Treatments with central stimulants initiated	Exacerbation of psychotic symptoms

This table illustrates key decision points in the clinical management of patients before contact with the Affective Disorders Clinic. It presents the interpretation of the clinical situation at the time, the action taken based on that interpretation, and the subsequent consequences of those actions.

**Table 2:** Key clinical recommendations and rationale for diagnosis and treatment in child and adolescent psychiatry.

Phase	Key clinical recommendations	Rationale
Diagnosis	Adhere to a hierarchical diagnostic approach during evaluations	Facilitates the identification of severe conditions, thereby reducing the duration of untreated episodes and mitigating elevated suicide risks
	Prioritize diagnostic utility in assessments	Accommodates the often non-specific and overlapping nature of symptoms during acute presentations, thereby optimizing treatment efficacy
	Integrate a longitudinal perspective into the diagnostic process	Allows differentiation of more persistent conditions, such as neurodevelopmental disorders, from treatable severe mental illness episodes, including psychotic and affective states
Treatment	Prioritize indicated medical interventions during acute disease states	Enables rapid symptom remission, minimizing exposure to untreated illness and facilitating subsequent psychosocial interventions
	Implement close follow-up and treatment for remission	Reveals latent symptoms following affective stabilization, facilitating complete recovery through comprehensive treatment until remission

This table outlines essential clinical recommendations and their underlying rationales in the diagnosis and treatment of psychiatric disorders in children and adolescents. Recommendations are divided into two main phases: diagnosis and treatment. Each phase contains key strategies accompanied by a rationale to optimize patient outcomes and facilitate clinical decision-making. The guidelines aim to improve the diagnostic accuracy, treatment efficacy, and long-term management of severe mental illnesses and neurodevelopmental disorders among the pediatric and adolescent populations.

**Table 3:** A summary of key affective and psychotic symptoms presented in each case.

	At initial presentation	Symtoms after initiation of first treatment
Patient 1	Depressive symptoms	Paranoid ideation
	Auditory hallucinations	Tactile hallucinations
		Experiences of thought reading
Patient 2	Atypical depression (hypersomnia, leaden paralysis, psychomotor retardation, pathological guilt, suicidality)	Auditory hallucinations in the absence of mood symptoms
	Mood-congruent auditory hallucinations	
Patient 3	Desorganized behavior	Behavioral cues of paranoia (preoccupation with window, cars outside)
	Desorganized speech	Verbalizations indicative of paranoia (pondering whether treating physician was police officer)
	Thought disorder	Persistence of disorganization of behavior and speech, attenuated over time with treatment
	Suddenly emerging highly aggressive behavior suggestive of paranoia	

A summary of key affective and psychotic symptoms present in each case. Patients 1 and 2 initially presented a clinical picture dominated by mood symptoms. Upon cessation of these symptoms, psychotic features independent of mood state became a more apparent part of the clinical picture. In the case of patient 3, psychotic symptoms were the dominant feature during the entirety of clinical contact.

### Supplementary information

Supplemental Material
