# Supplementary material for: Challenges and opportunities in the diagnosis and treatment of early-onset psychosis: a case series from the youth affective disorders clinic in Stockholm, Sweden
Source: Schizophrenia (Heidelb). 2024 Jan 3;10(1):5. doi: 10.1038/s41537-023-00427-z (PMC10851694; doi:10.1038/s41537-023-00427-z)
Supplement: Supplementary file 1 — Supplemental Material [file 41537_2023_427_MOESM1_ESM.docx]

**Supplemental Material**

**Table of contents**

**p. 2-3: Supplemental Table 1.**

Patient 1 – DSM-5 Criteria for Hypomanic Episodes and Major Depressive Disorder: Evaluation According to the LEAD Standard for Psychiatric Diagnosis

**p. 4-5: Supplemental Table 2.**

Patient 2 - DSM-5 Criteria for Hypomanic Episodes and Major Depressive Disorder: Evaluation According to the LEAD Standard for Psychiatric Diagnosis

**p. 6: Supplemental Text. 1**

Patient 3 – Disorganized Psychosis Diagnostic Elucidation

**p. 7-8: Supplemental Text. 2**

Patient perspectives

**Supplemental Table 1. Patient 1 – DSM-5 Criteria for Hypomanic Episodes and Major Depressive Disorder: Evaluation According to the LEAD Standard for Psychiatric Diagnosis**

|  |  |  |  |
| --- | --- | --- | --- |
|  | **Patient 1: Hypomanic Episode Checklist - Past Episode** |  |  |
|  |  |  |  |
|  | **Criteria** | **Symptoms (Yes/No)** |  |
|  | Duration | > 4 days |  |
|  | Elevated, expansive, or irritable mood | Yes |  |
|  | Inflated self-esteem or grandiosity | Yes |  |
|  | Decreased need for sleep | Yes |  |
|  | More talkative than usual or pressure to keep talking | Yes |  |
|  | Flight of ideas or subjective experience that thoughts are racing | Yes |  |
|  | Distractibility | Yes |  |
|  | Increase in goal-directed activity or psychomotor agitation | Yes |  |
|  | Excessive involvement in activities with high potential for negative consequences | No |  |
|  | Impact on Functioning | Yes |  |
|  | Exclusion due to substances/medical condition | No |  |
|  | Differentiation from Other Disorders | No |  |
|  | Number of episodes > 2 | Yes |  |
|  | Absence of Manic Episodes | Yes |  |
|  | This checklist synthesizes the assessment findings obtained through the Longitudinal, Expert, All Data (LEAD) standard, ensuring a robust and multi-faceted evaluation. It includes data from auto- and heteroanamnesis, self-rating questionnaires, structured clinical interviews, and thorough reviews of the patient's clinical history and psychiatric evaluations. Symptoms have been documented as present only when they signify a distinct and significant deviation from the individual's baseline behavior and are evaluated within the context of the youth's developmental stage and environmental circumstances. | |  |
|  |  |  |  |
|  | **Patient 1: Major Depressive Episode Checklist - Prior/Current Episode** | |  |
|  |  |  |  |
|  | **Criteria** | **Symptoms (Yes/No)** |  |
|  | Duration > 14 days | Yes |  |
|  | Depressed mood most of the day | Yes |  |
|  | Markedly diminished interest or pleasure in activities | Yes |  |
|  | Significant weight change or appetite disturbance | Yes |  |
|  | Insomnia or hypersomnia | Yes |  |
|  | Psychomotor agitation or retardation | Yes |  |
|  | Fatigue or loss of energy | Yes |  |
|  | Feelings of worthlessness or excessive guilt | Yes |  |
|  | Diminished ability to think or concentrate, or indecisiveness | Yes |  |
|  | Recurrent thoughts of death or suicidal ideation | Yes |  |
|  | Impact on Functioning | Yes |  |
|  | Exclusion due to substances/medical condition | No |  |
|  | Differentiation from Other Disorders | Yes |  |
|  | This checklist synthesizes the assessment findings obtained through the Longitudinal, Expert, All Data (LEAD) standard, ensuring a robust and multi-faceted evaluation. It includes data from auto- and heteroanamnesis, self-rating questionnaires, structured clinical interviews, and thorough reviews of the patient's clinical history and psychiatric evaluations. Symptoms have been documented as present only when they signify a distinct and significant deviation from the individual's baseline behavior and are evaluated within the context of the youth's developmental stage and environmental circumstances. | |  |
|  |  |  |  |

**Supplemental Table 2. Patient 2 - DSM-5 Criteria for Hypomanic Episodes and Major Depressive Disorder: Evaluation According to the LEAD Standard for Psychiatric Diagnosis**

|  |  |  |  |
| --- | --- | --- | --- |
|  | **Patient 2: Hypomanic Episode Checklist - Past Episode** |  |  |
|  |  |  |  |
|  | **Criteria** | **Symptoms (Yes/No)** |  |
|  | Duration | > 4 days |  |
|  | Elevated, expansive, or irritable mood | Yes |  |
|  | Inflated self-esteem or grandiosity | Yes |  |
|  | Decreased need for sleep | Yes |  |
|  | More talkative than usual or pressure to keep talking | Yes |  |
|  | Flight of ideas or subjective experience that thoughts are racing | Yes |  |
|  | Distractibility | Yes |  |
|  | Increase in goal-directed activity or psychomotor agitation | Yes |  |
|  | Excessive involvement in activities with high potential for negative consequences | Yes |  |
|  | Impact on Functioning | Yes |  |
|  | Exclusion due to substances/medical condition | No |  |
|  | Differentiation from Other Disorders | No |  |
|  | Number of episodes > 2 | Yes |  |
|  | Absence of Manic Episodes | Yes |  |
|  | This checklist synthesizes the assessment findings obtained through the Longitudinal, Expert, All Data (LEAD) standard, ensuring a robust and multi-faceted evaluation. It includes data from auto- and heteroanamnesis, self-rating questionnaires, structured clinical interviews, and thorough reviews of the patient's clinical history and psychiatric evaluations. Symptoms have been documented as present only when they signify a distinct and significant deviation from the individual's baseline behavior and are evaluated within the context of the youth's developmental stage and environmental circumstances. | |  |
|  |  |  |  |
|  | **Patient 2: Major Depressive Episode Checklist - Prior/Current Episode** | |  |
|  |  |  |  |
|  | **Criteria** | **Symptoms (Yes/No)** |  |
|  | Duration > 14 days | Yes |  |
|  | Depressed mood most of the day | Yes |  |
|  | Markedly diminished interest or pleasure in activities | Yes |  |
|  | Significant weight change or appetite disturbance | Yes |  |
|  | Insomnia or hypersomnia | Yes |  |
|  | Psychomotor agitation or retardation | Yes |  |
|  | Fatigue or loss of energy | Yes |  |
|  | Feelings of worthlessness or excessive guilt | Yes |  |
|  | Diminished ability to think or concentrate, or indecisiveness | Yes |  |
|  | Recurrent thoughts of death or suicidal ideation | Yes |  |
|  | Impact on Functioning | Yes |  |
|  | Exclusion due to substances/medical condition | No |  |
|  | Differentiation from Other Disorders | Yes |  |
|  | This checklist synthesizes the assessment findings obtained through the Longitudinal, Expert, All Data (LEAD) standard, ensuring a robust and multi-faceted evaluation. It includes data from auto- and heteroanamnesis, self-rating questionnaires, structured clinical interviews, and thorough reviews of the patient's clinical history and psychiatric evaluations. Symptoms have been documented as present only when they signify a distinct and significant deviation from the individual's baseline behavior and are evaluated within the context of the youth's developmental stage and environmental circumstances. | |  |
|  |  |  |  |

**Supplemental Text. Patient 3 – Disorganized Psychosis Diagnostic Elucidation**

The diagnosis of disorganized psychosis was approached with due consideration, grounded in a comprehensive evaluation of the patient's symptoms which were incongruent with his baseline functioning. The patient's decline was not solely characterized by aggression and restlessness, typical of his developmental disorders, but also by a notable deterioration in organized thought and language—a pivotal aspect of disorganized psychosis. The disorganized speech observed, exemplified by the patient's irrelevant remarks about police cars, signified a disruption in thought processes beyond what could be attributed to intellectual disability or autism.

While Risperidone is indeed beneficial for a spectrum of behavioral issues in children with intellectual disabilities, the marked improvement in symptoms of disorganization following its administration suggests a specific antipsychotic response. The patient's positive reaction to Risperidone was distinct from the general behavioral modulations often seen in children treated for aggression or irritability associated with autism. This was particularly evident in the amelioration of thought disorganization, which is not a typical domain of improvement with Risperidone in cases of intellectual disability alone.

Furthermore, the careful cessation of central stimulants, which could exacerbate symptoms of psychosis, coupled with the patient's history and familial background, reinforces the diagnosis. The therapeutic strategy, including the initiation and careful titration of Risperidone, was based on a holistic assessment of the patient's unique presentation, rather than a generalized approach to symptom management.

The observed clinical improvements were consistent with the known effects of Risperidone on psychotic symptoms rather than just behavioral symptoms associated with intellectual disability. This nuanced understanding underscores our rationale for the diagnosis of disorganized psychosis, rather than attributing the improvements solely to the effects of Risperidone on behavioral challenges in intellectual disability.

**Patient perspectives**

**Patient 1**

The patient and her caregivers report a profound improvement in her quality of life, crediting the affective stability provided by the treatment regimen of lithium, risperidone, and olanzapine. This newfound equilibrium has been pivotal in fostering her social engagements and participation in family activities, such as vacations, that were once not feasible. Her reinvigorated involvement in karate and enhanced self-care for her diabetes are also attributed to this stable emotional baseline. Although there is a recognition of the appetite increase and weight gain associated with her medications, the patient and her caregivers believe that the emotional steadiness she now enjoys has empowered her to proactively pursue lifestyle modifications to mitigate these side effects.

**Patient 2**

The family collectively experiences a significant uplift in their quality-of-life post-treatment. They find joy in enriched family dynamics and the normalization of life as collective attention has shifted away from the patient’s psychiatric condition towards more fulfilling familial connections and shared activities. The patient has successfully re-engaged with her social circle and hobbies, which has been a source of personal rejuvenation. The parents particularly appreciate the marked decrease in their caregiving demands. While the treatment initially introduced increased appetite and weight gain, the patient is proactively managing these through lifestyle adjustments. Notably, the initial fatigue encountered with the treatment was transient and abated with the optimization of risperidone dosage. Being informed about the possibility of such side effects in advance mitigated concerns, allowing the patient to focus on the positive aspects of her recovery journey.

**Patient 3**

Post-treatment, there has been a remarkable enhancement in the family's dynamic, with the mother and son now able to engage in activities together independently, fostering a stronger bond. The ease of communication with the son has significantly improved, a change that has been deeply appreciated by the parents, alongside notable advancements in his language skills. The son continues to require support for his neuropsychiatric condition, yet there's a clear and commendable increase in his functional capabilities – completing homework with less assistance, maintaining regular school attendance, and enjoying interactions with peers are all testaments to his progress. Although there is an acknowledgment of some psychological discomfort due to the medication, this is an area of ongoing attention and care.
